# 1,2-Bis{2-[2-(trimethyl­sil­yl)ethyn­yl]phen­yl}ethane-1,2-dione

**DOI:** 10.1107/S160053681203663X

**Published:** 2012-08-31

**Authors:** Christopher R. Sparrow, Frank R. Fronczek, Steven F. Watkins

**Affiliations:** a Department of Chemistry, Louisiana State University, Baton Rouge, LA 70803-1804, USA

## Abstract

The title compound, C_24_H_26_O_2_Si_2_, has *C*_2_ crystallographic symmetry. The dihedral angle between the aromatic rings is 84.5 (2)°. The acetyl­ene group is slightly non-linear, with angles at the acetyl­ene C atoms of 175.7 (2) and 177.0 (2)°. In the crystal structure, only van de Waals interactions occur.

## Related literature

For the structure of benzil, see Brown & Sadanaga (1965 ▶); Gabe *et al.* (1981 ▶); More *et al.* (1987 ▶). For the synthesis see: Garcia *et al.* (1995 ▶). For the determination of absolute configuration from Bijvoet pairs, see: Hooft *et al.* (2008 ▶).
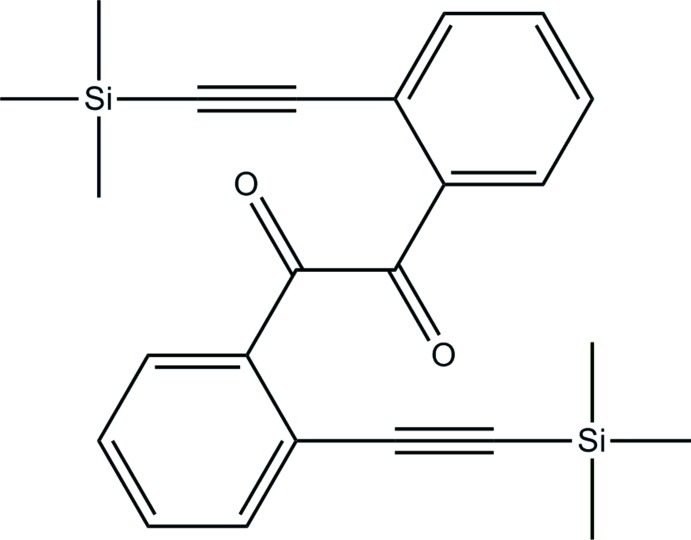


## Experimental

### 

#### Crystal data

C_24_H_26_O_2_Si_2_
*M* *_r_* = 402.63
Trigonal, 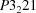

*a* = 9.2241 (1) Å
*c* = 23.7787 (5) Å
*V* = 1752.13 (5) Å^3^
*Z* = 3
Mo *K*α radiation
μ = 0.17 mm^−1^
*T* = 120 K
0.25 × 0.25 × 0.25 mm

#### Data collection

Nonius KappaCCD diffractometer
Absorption correction: multi-scan (*SCALEPACK*; Otwinowski & Minor, 1997 ▶) *T*_min_ = 0.959, *T*_max_ = 0.959
23012 measured reflections
3410 independent reflections
2325 reflections with *I* > 2σ(*I*)
*R*_int_ = 0.047

#### Refinement

*R*[*F* ^2^ > 2σ(*F* ^2^)] = 0.043
*wR*(*F* ^2^) = 0.103
*S* = 1.00
3410 reflections
131 parameters
H-atom parameters constrained
Δρ_max_ = 0.25 e Å^−3^
Δρ_min_ = −0.21 e Å^−3^
Absolute structure: Flack (1983 ▶), 1419 Bijvoet pairs
Flack parameter: 0.0 (1)

### 

Data collection: *COLLECT* (Nonius, 2000 ▶); cell refinement: *DENZO* and *SCALEPACK* (Otwinowski & Minor, 1997 ▶); data reduction: *DENZO* and *SCALEPACK*; program(s) used to solve structure: *SIR2002* (Burla *et al.*, 2003 ▶); program(s) used to refine structure: *SHELXL97* (Sheldrick, 2008 ▶); molecular graphics: *ORTEP-3 for Windows* (Farrugia, 1997 ▶); software used to prepare material for publication: *WinGX* (Farrugia, 1999 ▶).

## Supplementary Material

Crystal structure: contains datablock(s) global, I. DOI: 10.1107/S160053681203663X/bx2422sup1.cif

Structure factors: contains datablock(s) I. DOI: 10.1107/S160053681203663X/bx2422Isup2.hkl

Supplementary material file. DOI: 10.1107/S160053681203663X/bx2422Isup3.cml

Additional supplementary materials:  crystallographic information; 3D view; checkCIF report

## Figures and Tables

**Fig. 1.**
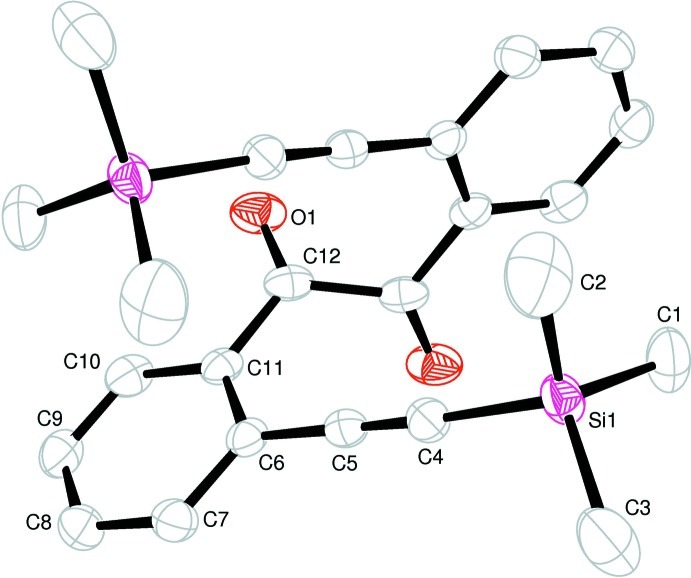
View of (I) (50% probability displacement ellipsoids)

## supplementary crystallographic information

### Comment

The title compound (I), (C_12_H_13_OSi)_2_, lies on a crystallographic twofold axis. The phenyl ring is planar - all six C atoms have δ/σ < 0.2. However, carbonyl carbon C12 is 0.217 (5) Å above the C11—O1—C12' plane, and the C10—C11—C12—O1 torsion angle is 19.5 (3)°. The ethanedione C12(*sp*^2^)—C12(*sp*^2^)' distance of 1.538 (4) Å is somewhat longer than expected, but is consistent with values reported for benzil, which average 1.536 (10) Å. The acetylenic moiety is non-linear with deviations from a weighted least-squares line of δ(Si1) = 0.0034 (15), δ(C4) = 0.054 (4), δ(C5) = 0.049 (4), and δ(C6) = 0.047 (4) Å. The crystal structure is stablized by van der Waals interactions.

### Experimental

The title compound was supplied by J. Gabriel Garcia, having been synthesized from 1,2-bis-(2-bromophenyl)-ethane-1,2-dione and trimethylsilyl acetylene (Garcia *et al.*, 1995).

### Refinement

The space group assignment and absolute structure are based on analysis of 1419 Bijvoet pairs, Flack (1983) parameter *x* = 0.0 (1), Hooft *et al.* (2008) parameter *y* = -0.04 (7), and Hooft P2(true) = 1.000.

All H atoms were placed in calculated positions with C—H distances of 0.95 (aromatic) and 0.98 Å (methyl) and *U*_iso_ = 1.2 or 1.5 *U*_eq_ of the attached *sp*^2^ or *sp*^3^ C atom, and thereafter treated as riding. A torsional parameter was refined for each methyl group.

### Crystal data

**Table:**

C_24_H_26_O_2_Si_2_	*D*_x_ = 1.145 Mg m^−^^3^
*M**_r_* = 402.63	Mo *K*α radiation, λ = 0.71073 Å
Trigonal, *P*3_2_21	Cell parameters from 3424 reflections
Hall symbol: P 32 2"	θ = 2.5–30.0°
*a* = 9.2241 (1) Å	µ = 0.17 mm^−^^1^
*c* = 23.7787 (5) Å	*T* = 120 K
*V* = 1752.13 (5) Å^3^	Rhombohedron, yellow
*Z* = 3	0.25 × 0.25 × 0.25 mm
*F*(000) = 642	

### Data collection

**Table:**

Nonius KappaCCD diffractometer	3410 independent reflections
Radiation source: sealed tube	2325 reflections with *I* > 2σ(*I*)
Horizonally mounted graphite crystal monochromator	*R*_int_ = 0.047
Detector resolution: 9 pixels mm^-1^	θ_max_ = 30.0°, θ_min_ = 2.6°
ω and φ scans	*h* = −12→12
Absorption correction: multi-scan (*SCALEPACK*; Otwinowski & Minor, 1997)	*k* = −10→10
*T*_min_ = 0.959, *T*_max_ = 0.959	*l* = −31→33
23012 measured reflections	

### Refinement

**Table:**

Refinement on *F*^2^	Secondary atom site location: difference Fourier map
Least-squares matrix: full	Hydrogen site location: inferred from neighbouring sites
*R*[*F*^2^ > 2σ(*F*^2^)] = 0.043	H-atom parameters constrained
*wR*(*F*^2^) = 0.103	*w* = 1/[σ^2^(*F*_o_^2^) + (0.0519*P*)^2^] where *P* = (*F*_o_^2^ + 2*F*_c_^2^)/3
*S* = 1.00	(Δ/σ)_max_ < 0.001
3410 reflections	Δρ_max_ = 0.25 e Å^−^^3^
131 parameters	Δρ_min_ = −0.21 e Å^−^^3^
0 restraints	Absolute structure: Flack (1983), 1419 Bijvoet pairs
0 constraints	Flack parameter: 0.0 (1)
Primary atom site location: structure-invariant direct methods	

### Special details

**Table:**

Geometry. All e.s.d.'s (except the e.s.d. in the dihedral angle between two l.s. planes) are estimated using the full covariance matrix. The cell e.s.d.'s are taken into account individually in the estimation of e.s.d.'s in distances, angles and torsion angles; correlations between e.s.d.'s in cell parameters are only used when they are defined by crystal symmetry. An approximate (isotropic) treatment of cell e.s.d.'s is used for estimating e.s.d.'s involving l.s. planes.

### Fractional atomic coordinates and isotropic or equivalent isotropic displacement parameters (Å^2^)

**Table:**

	*x*	*y*	*z*	*U*_iso_*/*U*_eq_	
C1	0.4411 (3)	−0.1681 (3)	0.00979 (9)	0.0487 (6)	
H1A	0.5479	−0.0985	−0.0095	0.073*	
H1B	0.3539	−0.233	−0.018	0.073*	
H1C	0.4522	−0.2447	0.0357	0.073*	
C2	0.1985 (3)	−0.1571 (4)	0.09491 (10)	0.0714 (8)	
H2A	0.2222	−0.2247	0.1211	0.107*	
H2B	0.1022	−0.2309	0.0714	0.107*	
H2C	0.1731	−0.0815	0.1162	0.107*	
C3	0.3475 (4)	0.1018 (3)	0.00053 (11)	0.0802 (10)	
H3A	0.3209	0.1763	0.0219	0.12*	
H3B	0.2539	0.0314	−0.0245	0.12*	
H3C	0.4487	0.1688	−0.0219	0.12*	
C4	0.5616 (2)	0.1008 (2)	0.09647 (7)	0.0310 (4)	
C5	0.6750 (2)	0.1814 (2)	0.12846 (7)	0.0282 (4)	
C6	0.8026 (2)	0.2815 (2)	0.16901 (6)	0.0283 (4)	
C7	0.8414 (2)	0.4474 (2)	0.17783 (8)	0.0346 (5)	
H7	0.7881	0.4931	0.1558	0.042*	
C8	0.9559 (2)	0.5446 (2)	0.21811 (8)	0.0385 (5)	
H8	0.982	0.6572	0.2233	0.046*	
C9	1.0332 (2)	0.4800 (2)	0.25096 (7)	0.0381 (5)	
H9	1.1116	0.5479	0.2789	0.046*	
C10	0.9970 (2)	0.3169 (2)	0.24337 (7)	0.0352 (4)	
H10	1.0497	0.2725	0.2664	0.042*	
C11	0.8830 (2)	0.2166 (2)	0.20188 (7)	0.0274 (4)	
C12	0.8518 (2)	0.0429 (2)	0.19563 (7)	0.0309 (4)	
O1	0.88376 (18)	−0.02847 (17)	0.23231 (6)	0.0457 (4)	
Si1	0.38331 (7)	−0.03278 (7)	0.04974 (2)	0.03309 (15)	

### Atomic displacement parameters (Å^2^)

**Table:**

	*U*^11^	*U*^22^	*U*^33^	*U*^12^	*U*^13^	*U*^23^
C1	0.0621 (15)	0.0523 (14)	0.0410 (11)	0.0355 (13)	−0.0166 (11)	−0.0167 (10)
C2	0.0331 (13)	0.092 (2)	0.0657 (15)	0.0134 (14)	−0.0059 (12)	−0.0155 (14)
C3	0.108 (2)	0.0446 (14)	0.0940 (18)	0.0423 (15)	−0.0758 (18)	−0.0214 (13)
C4	0.0351 (10)	0.0292 (10)	0.0311 (9)	0.0178 (9)	−0.0057 (8)	−0.0005 (8)
C5	0.0316 (10)	0.0258 (9)	0.0288 (9)	0.0156 (8)	−0.0008 (8)	0.0041 (8)
C6	0.0246 (9)	0.0290 (10)	0.0243 (8)	0.0083 (8)	0.0013 (7)	0.0042 (8)
C7	0.0346 (11)	0.0295 (11)	0.0344 (10)	0.0120 (9)	−0.0044 (8)	0.0022 (8)
C8	0.0361 (12)	0.0273 (11)	0.0401 (11)	0.0068 (10)	0.0020 (9)	−0.0008 (8)
C9	0.0256 (10)	0.0386 (11)	0.0312 (9)	0.0019 (9)	−0.0042 (8)	−0.0033 (8)
C10	0.0233 (9)	0.0403 (11)	0.0339 (9)	0.0098 (9)	−0.0013 (8)	0.0075 (8)
C11	0.0194 (8)	0.0302 (9)	0.0260 (8)	0.0075 (8)	0.0033 (7)	0.0069 (7)
C12	0.0196 (9)	0.0329 (10)	0.0361 (10)	0.0101 (8)	0.0025 (8)	0.0105 (8)
O1	0.0440 (9)	0.0402 (9)	0.0499 (8)	0.0188 (8)	−0.0108 (7)	0.0125 (7)
Si1	0.0354 (3)	0.0283 (3)	0.0372 (3)	0.0172 (3)	−0.0128 (2)	−0.0072 (2)

### Geometric parameters (Å, º)

**Table:**

C1—Si1	1.847 (2)	C5—C6	1.443 (2)
C1—H1A	0.98	C6—C7	1.403 (3)
C1—H1B	0.98	C6—C11	1.401 (3)
C1—H1C	0.98	C7—C8	1.375 (3)
C2—Si1	1.849 (2)	C7—H7	0.95
C2—H2A	0.98	C8—C9	1.378 (3)
C2—H2B	0.98	C8—H8	0.95
C2—H2C	0.98	C9—C10	1.380 (3)
C3—Si1	1.852 (2)	C9—H9	0.95
C3—H3A	0.98	C10—C11	1.401 (2)
C3—H3B	0.98	C10—H10	0.95
C3—H3C	0.98	C11—C12	1.487 (3)
C4—C5	1.203 (2)	C12—O1	1.214 (2)
C4—Si1	1.8522 (19)	C12—C12^i^	1.538 (4)
			
Si1—C1—H1A	109.5	C8—C7—H7	119.7
Si1—C1—H1B	109.5	C6—C7—H7	119.7
H1A—C1—H1B	109.5	C9—C8—C7	120.52 (19)
Si1—C1—H1C	109.5	C9—C8—H8	119.7
H1A—C1—H1C	109.5	C7—C8—H8	119.7
H1B—C1—H1C	109.5	C8—C9—C10	120.12 (17)
Si1—C2—H2A	109.5	C8—C9—H9	119.9
Si1—C2—H2B	109.5	C10—C9—H9	119.9
H2A—C2—H2B	109.5	C9—C10—C11	120.31 (17)
Si1—C2—H2C	109.5	C9—C10—H10	119.8
H2A—C2—H2C	109.5	C11—C10—H10	119.8
H2B—C2—H2C	109.5	C6—C11—C10	119.58 (17)
Si1—C3—H3A	109.5	C6—C11—C12	123.09 (16)
Si1—C3—H3B	109.5	C10—C11—C12	117.32 (17)
H3A—C3—H3B	109.5	O1—C12—C11	122.93 (17)
Si1—C3—H3C	109.5	O1—C12—C12^i^	115.72 (18)
H3A—C3—H3C	109.5	C11—C12—C12^i^	120.33 (17)
H3B—C3—H3C	109.5	C3—Si1—C1	109.67 (11)
C5—C4—Si1	177.01 (16)	C3—Si1—C2	111.37 (14)
C4—C5—C6	175.7 (2)	C1—Si1—C2	111.58 (13)
C7—C6—C11	118.82 (16)	C3—Si1—C4	109.27 (10)
C7—C6—C5	118.66 (17)	C1—Si1—C4	107.33 (9)
C11—C6—C5	122.44 (17)	C2—Si1—C4	107.49 (9)
C8—C7—C6	120.63 (18)		
			
C11—C6—C7—C8	−0.2 (3)	C9—C10—C11—C6	−1.6 (3)
C5—C6—C7—C8	176.61 (16)	C9—C10—C11—C12	179.24 (16)
C6—C7—C8—C9	−0.7 (3)	C6—C11—C12—O1	−159.64 (18)
C7—C8—C9—C10	0.5 (3)	C10—C11—C12—O1	19.5 (3)
C8—C9—C10—C11	0.7 (3)	C6—C11—C12—C12^i^	32.4 (2)
C7—C6—C11—C10	1.3 (2)	C10—C11—C12—C12^i^	−148.44 (13)
C5—C6—C11—C10	−175.35 (16)	C11—C12—C12^i^—C11^i^	−132.9 (2)
C7—C6—C11—C12	−179.56 (16)	C11—C12—C12^i^—O1^i^	58.36 (11)
C5—C6—C11—C12	3.8 (3)	O1—C12—C12^i^—O1^i^	−110.4 (3)

Symmetry code: (i) *x*−*y*, −*y*, −*z*+1/3.
